# Prediction of Left Ventricular Systolic Dysfunction Using an Artificial Intelligence-Based Electrocardiogram Analysis Model in Patients Presenting to the Emergency Department

**DOI:** 10.3390/diagnostics16162587

**Published:** 2026-08-15

**Authors:** Mi Jin Lee, Haewon Jung

**Affiliations:** 1 Department of Emergency Medicine, The Catholic University of Korea St. Vincent’s Hospital, Suwon 16247, Republic of Korea; 2 Department of Emergency Medicine, School of Medicine, Kyungpook National University, Daegu 41944, Republic of Korea

**Keywords:** artificial intelligence, electrocardiogram, emergency department, left ventricular dysfunction

## Abstract

**Background:** Left ventricular systolic dysfunction (LVSD) is a precursor to heart failure arising from diverse cardiac conditions. Although echocardiography remains the reference standard for LVSD diagnosis, its routine use in the emergency department (ED) may be constrained by cost, time, equipment availability, and the need for specialized expertise. We evaluated the diagnostic performance of an artificial intelligence-based electrocardiogram analysis model (AI-ECG model) for detecting LVSD in patients presenting to the ED. **Methods**: This retrospective observational study included patients treated at a single tertiary hospital between 2020 and 2022 who underwent 12-lead electrocardiography within 24 h of ED admission and echocardiography within 30 days. Electrocardiographic data were analyzed using AiTiALVSD version 1.00.00, with a predefined cutoff score of 9.7 used to classify patients as being at high or low risk of LVSD. Diagnostic performance was assessed using standard discrimination and classification metrics. **Results**: Among 4529 included patients, 531 had LVSD. The AI-ECG model demonstrated high discrimination, with an area under the receiver operating characteristic curve (AUROC) of 0.934 (95% confidence interval [CI]: 0.923–0.945). Performance remained robust in patients with a shock index ≥ 0.9 (*n* = 453; AUROC, 0.895; 95% CI: 0.854–0.937) and in those with hypotension (*n* = 75; AUROC, 0.885; 95% CI: 0.786–0.984). **Conclusions**: The AI-ECG model accurately identified LVSD in ED patients in this cohort despite heterogeneous acquisition conditions and retained good discrimination in hemodynamically unstable subgroups, although findings in the smaller hypotensive subgroup should be interpreted as exploratory.

## 1. Introduction

Left ventricular systolic dysfunction (LVSD) is an important precursor to heart failure and may arise from diverse etiologies, including cardiomyopathy, myocardial infarction, and structural cardiac abnormalities [1]. Echocardiography remains the standard modality for identifying LVSD; however, its cost, dependence on specialized equipment and expertise, and time-intensive nature may limit its universal application [2]. By contrast, electrocardiography (ECG) offers a more accessible and rapid alternative [2,3]. Building on these practical advantages, numerous studies have investigated the use of artificial intelligence (AI) algorithms to detect LVSD from ECG data [4,5,6,7].

Even in asymptomatic individuals, LVSD is associated with an increased risk of progression to congestive heart failure and a corresponding increase in mortality, underscoring the importance of timely detection and intervention [8,9]. In the emergency department (ED), echocardiographic assessment may be particularly challenging because of fluctuating clinical conditions, limitations in image acquisition and interpretation, and substantial time constraints [10]. Moreover, although previous studies of ECG-based LVSD detection have predominantly included clinically stable populations, the model’s performance in routine emergency care remains uncertain [11]. Patients in the ED commonly present with acute pain, agitation, respiratory distress, and other forms of physiological instability, all of which may complicate ECG acquisition and potentially compromise AI-based analysis.

Since the present cohort was assembled, the evidence supporting AI-ECG detection of LVSD has expanded substantially. The algorithm evaluated in this study has subsequently been validated against echocardiography in cohorts of patients with suspected LVSD, demonstrating discrimination comparable to that reported previously, and AI-ECG screening has been extended to additional settings and populations, including resource-limited primary care environments [12,13]. Nevertheless, these evaluations have largely involved clinically stable or elective populations. The performance of the algorithm in hemodynamically unstable patients presenting to the ED, particularly those with hypotension or an elevated shock index, has not been specifically assessed, even though rapid and reliable evaluation of left ventricular function may directly inform urgent therapeutic decisions such as fluid resuscitation.

Accordingly, the present study evaluated an AI-based ECG analysis model (AI-ECG model; AiTiALVSD, Medical AI Co., Ltd., Seoul, Republic of Korea) for detecting LVSD in patients presenting to the ED, including those with abnormal vital signs.

## 2. Materials and Methods

### 2.1. Study Design, Setting, and Population

This retrospective observational study was conducted at a single tertiary care hospital that receives more than 30,000 ED visits annually. Patients were eligible if they presented to the ED between 2020 and 2022, underwent 12-lead electrocardiography within 24 h of ED admission, and underwent echocardiography with documented left ventricular ejection fraction (LVEF) within 30 days of admission. Patients younger than 18 years and those presenting with injury-related conditions were excluded. The Institutional Review Board of Kyungpook National University Hospital approved the study and waived the requirement for informed consent because of its retrospective design.

### 2.2. Study Protocol

Raw electrocardiographic data were acquired using a MAC 3500 resting ECG system (GE Healthcare, Chicago, IL, USA) and extracted from the MUSE ECG data management system server (GE Healthcare, Chicago, IL, USA). When multiple ECGs were obtained following an ED visit, the first recorded ECG was used. LVEF was determined from two-dimensional echocardiography using the Simpson method, and LVSD was defined as an echocardiographic LVEF ≤ 40%. Patients who underwent echocardiography but had no documented ejection fraction in the formal report were excluded. For patients with multiple echocardiographic examinations, the result obtained closest to ED admission was used.

Vital signs and chief complaints were obtained from the initial triage documentation entered into the electronic medical records by the emergency medical technician at the time of the ED visit. Chief complaints were classified into 20 categories according to version 1.1 of the Canadian Emergency Department Information System (CEDIS), with all categories other than the four most frequent grouped as “Other” [14]. For patients presenting with cardiac arrest, vital signs were recorded after resuscitation and the return of spontaneous circulation. Weight, height, and medical history, including hypertension, diabetes mellitus, cardiovascular disease, chronic kidney disease, and stroke, were obtained from nursing assessment records entered into the electronic medical records. Cardiovascular disease was defined as a previous diagnosis of coronary artery disease, myocardial infarction, pulmonary embolism, cardiomyopathy, valvular heart disease, atrial or ventricular arrhythmia, heart failure, or pericardial disease. The first N-terminal pro-B-type natriuretic peptide (NT-proBNP) measurement and chest radiography result recorded within 24 h after the ED visit were included in the analysis; data recorded beyond this period were excluded. NT-proBNP was quantified using the Atellica IM B-type natriuretic peptide assay on an Atellica IM analyzer (Siemens Healthineers, Malvern, PA, USA). Chest radiography findings were based on formal interpretations by radiologists. Solitary nodules, old tuberculosis lesions, and other findings considered inactive pulmonary lesions were not classified as abnormal chest radiographic findings. ED diagnoses were categorized as “heart disease” or “non-heart disease” according to International Classification of Diseases, Tenth Revision (ICD-10) codes. Heart disease included I20–I25 (ischemic heart diseases), I00–I09 (rheumatic heart diseases), and I30–I39 (other forms of heart disease); all remaining diagnoses were classified as non-heart disease. The admission-to-echocardiography interval was calculated from the time of ED admission. All study data were obtained from the clinical data warehouse of Kyungpook National University Hospital.

### 2.3. AI/Machine Learning (ML)-Based Software as a Medical Device Using 12-Lead ECG

In 2019, Kwon et al. introduced an AI model for LVSD screening using 12-lead ECG data [15]. This work culminated in 2023 in the development of an AI/machine learning-based software as a medical device (SaMD), which was subsequently cleared by the Ministry of Food and Drug Safety of the Republic of Korea. The resulting software, AiTiALVSD version 1.00.00, analyzes 12-lead ECG data to screen for LVSD. The algorithm was developed using several hundred thousand 12-lead ECGs paired with transthoracic echocardiographic findings collected between February 1996 and June 2021 at tertiary and cardiac specialty hospitals in the Republic of Korea. ECGs were acquired using GE and Philips systems, and the raw signals were sampled at 500 Hz. AiTiALVSD was developed using a residual neural network architecture, as described previously [5,16]. The model uses digital signals from 12-lead ECG systems as its sole input and does not incorporate additional clinical variables. The software generates an LVSD probability score ranging from 0 to 100, reported as a decimal value rounded to one decimal place. A predefined score of 9.7 was used to distinguish high- from low-risk results. This threshold was selected to achieve approximately 90% sensitivity in the derivation dataset, consistent with the operating-point convention adopted during the development of this class of algorithms [3,15] and reflecting the model’s intended use as a screening rather than a diagnostic tool. Kyungpook National University Hospital contributed no electrocardiographic or echocardiographic data to algorithm development [3,15]. AiTiALVSD version 1.00.00 had been finalized before initiation of the present study and was applied without modification or retraining. The present study therefore constitutes an external validation in a cohort independent of the development dataset.

### 2.4. Outcome Measures

The primary outcome was the performance of the AI-ECG model in detecting LVSD among patients presenting to the ED, using echocardiographically determined LVEF as the reference standard. The secondary outcome was model performance in subgroups defined by abnormal vital signs, specifically hypotension, defined as systolic blood pressure (SBP) < 90 mmHg, and a shock index ≥ 0.9, based on the conventional threshold of 0.9 [17]. When ECG data are entered into the AI-ECG model, the algorithm generates a score representing the likelihood of an LVEF ≤ 40%. LVSD risk was classified as “high” when the score was ≥ 9.7 and “low” when the score was below 9.7; this cutoff was selected out of an emphasis on sensitivity.

### 2.5. Statistical Analysis

Continuous variables are presented as medians [interquartile ranges], and categorical variables are presented as frequencies and proportions. Baseline characteristics were compared using the Mann–Whitney U test for continuous variables and the chi-square test for categorical variables. The diagnostic performance of the AI-ECG model was evaluated for both the primary and secondary outcomes using the area under the receiver operating characteristic curve (AUROC), area under the precision–recall curve (AUPRC), sensitivity, specificity, positive predictive value (PPV), and negative predictive value (NPV), with each estimate reported alongside its 95% confidence interval (CI). The continuous LVSD probability score was used to calculate the AUROC and AUPRC, whereas the predefined cutoff of 9.7 was applied to calculate sensitivity, specificity, PPV, and NPV. A *p*-value < 0.05 was considered statistically significant. All analyses were performed using R version 4.1.0 (R Foundation for Statistical Computing, Vienna, Austria) and Python version 3.8 (Python Software Foundation, Wilmington, DE, USA).

## 3. Results

### 3.1. Study Population and Baseline Characteristics

**Figure 1:**
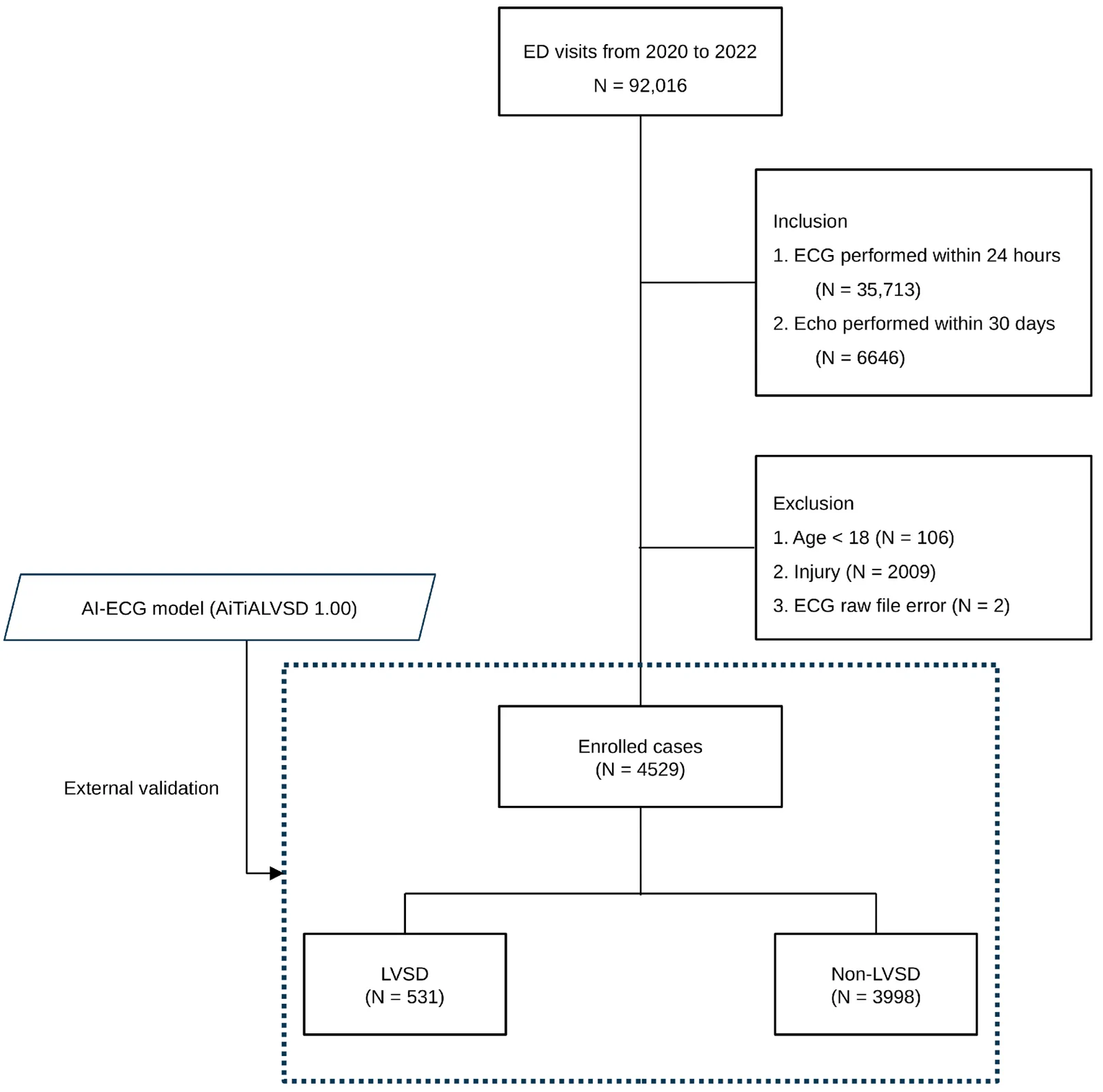
Flowchart of participant inclusion. AI-ECG, artificial intelligence-based electrocardiogram; ECG, electrocardiogram; ED, emergency department; LVSD, left ventricular systolic dysfunction.

**Figure 2:**
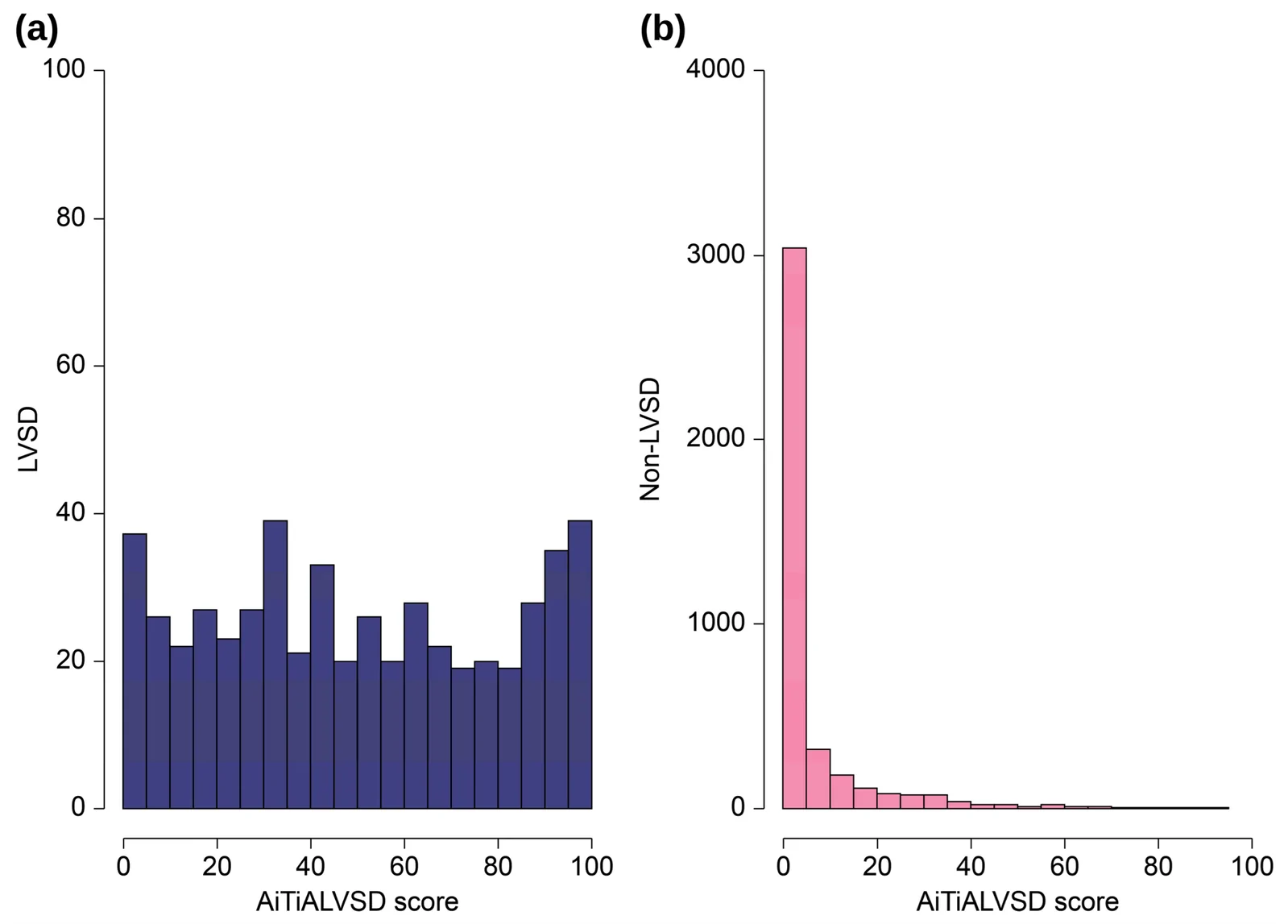
Distribution of AI-ECG model (AiTiALVSD) scores according to left ventricular systolic function. (**a**) Patients with LVSD (*n* = 531). (**b**) Patients without LVSD (*n* = 3998). The y-axis indicates the number of patients within each score interval. LVSD, left ventricular systolic dysfunction.

During the study period, 92,016 patients presented to the ED, of whom 35,713 (38.8%) underwent 12-lead electrocardiography within 24 h of arrival. Among these patients, 6646 (18.6%) underwent echocardiography with a documented ejection fraction within 30 days. After excluding patients younger than 18 years (*n* = 106), those presenting with injury-related conditions (*n* = 2009), and those with an ECG raw-file error (*n* = 2), 4529 patients were included in the analysis (Figure 1). Of these, 531 patients (11.7%) had LVSD, defined as an LVEF ≤ 40%, whereas 3998 did not have LVSD (Table 1). Diabetes mellitus, cardiovascular disease, and chronic kidney disease were more prevalent among patients with LVSD than among those without LVSD (all *p* < 0.001). Heart rate, respiratory rate, and body temperature also differed significantly between the groups (all *p* < 0.001). Abnormal chest radiographic findings were substantially more frequent in the LVSD group than in the non-LVSD group (54.9% vs. 21.8%, respectively). AI-ECG model scores also differed markedly between the groups, with substantially higher scores among patients with LVSD (Figure 2).

The distribution of chief complaints differed significantly between the groups. Respiratory complaints were more common among patients with LVSD than among those without LVSD (40.9% vs. 11.4%), whereas neurologic complaints were more frequent among patients without LVSD (28.4% vs. 4.7%).

Echocardiography was performed on the day of ED admission in 883 patients (19.5%), within 3 days in 3071 patients (67.8%), and within 7 days in 3924 patients (86.6%); 605 patients (13.4%) underwent echocardiography more than 7 days after admission. The admission-to-echocardiography interval was shorter among patients with LVSD than among those without LVSD (median, 1.0 vs. 2.0 days; *p* < 0.001), and 81.4% of patients with LVSD underwent echocardiography within 3 days.

### 3.2. Performance of the AI-ECG Model

**Figure 3:**
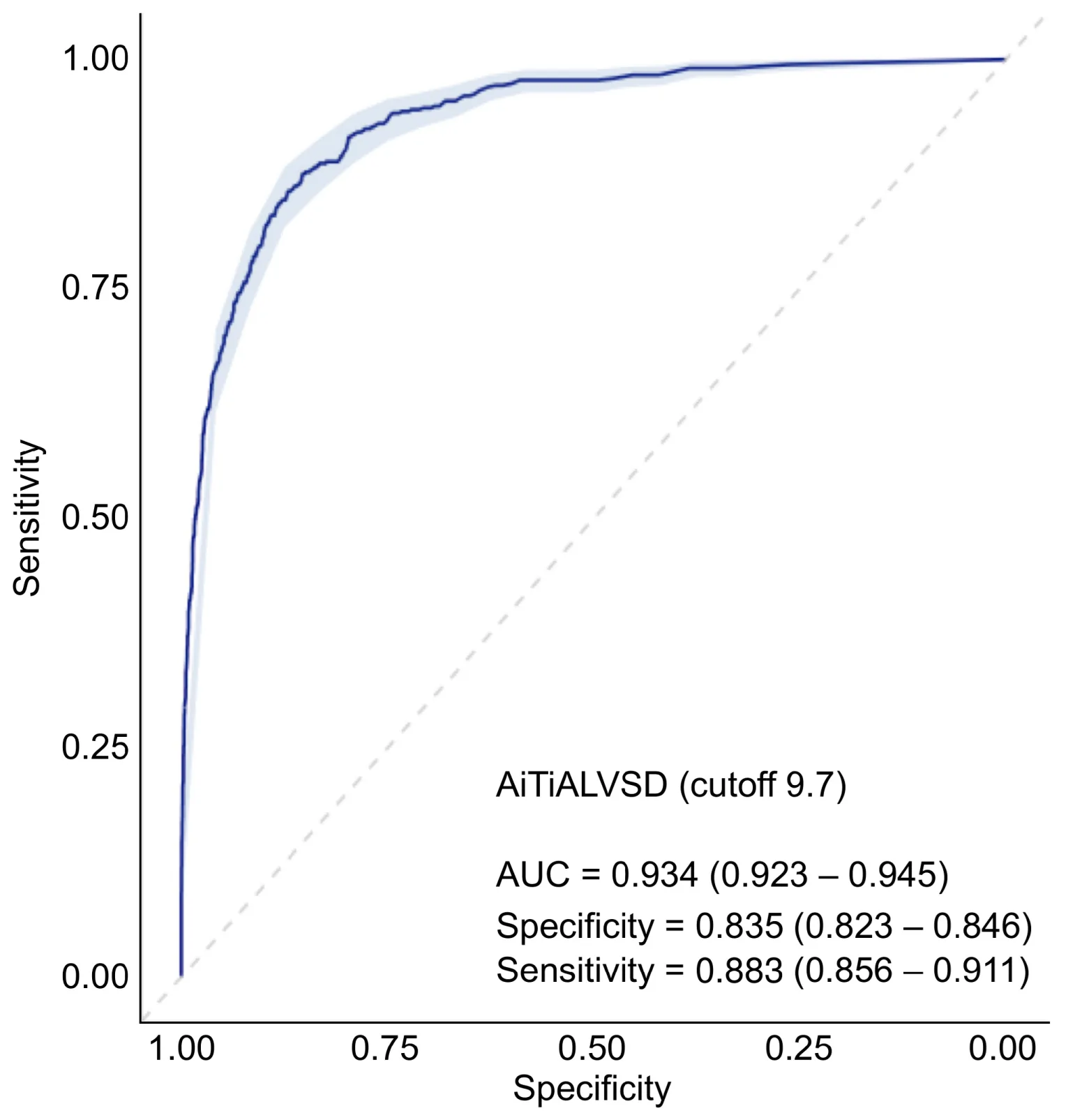
Receiver operating characteristic curve of the AI-ECG model (AiTiALVSD) for detecting left ventricular systolic dysfunction. AUC, area under the curve.

The diagnostic performance of the AI-ECG model is summarized in Table 2. The model demonstrated high discrimination, with an AUROC of 0.934 (95% CI: 0.923–0.945) (Figure 3) and an AUPRC of 0.735 (95% CI: 0.661–0.754). At the predefined cutoff, sensitivity was 0.883 (95% CI: 0.856–0.911), specificity was 0.835 (95% CI: 0.823–0.846), PPV was 0.415 (95% CI: 0.386–0.444), and NPV was 0.982 (95% CI: 0.977–0.986).

### 3.3. Performance of the AI-ECG Model in Patients with Hypotension and a Shock Index ≥ 0.9

The diagnostic performance of the AI-ECG model was further evaluated in subgroups defined by vital signs, as summarized in Table 3. Systolic blood pressure was not recorded at triage in 34 patients, who were therefore excluded from the subgroup analyses. Among patients with an SBP < 90 mmHg (*n* = 75), the model yielded an AUROC of 0.885 (95% CI: 0.786–0.984) and an AUPRC of 0.633 (95% CI: 0.383–0.843). Sensitivity was 0.900 (95% CI: 0.714–1.00), specificity was 0.708 (95% CI: 0.597–0.818), PPV was 0.321 (95% CI: 0.148–0.494), and NPV was 0.979 (95% CI: 0.937–1.00). Among patients with a shock index ≥ 0.9 (*n* = 453), including 103 with LVSD, the model achieved an AUROC of 0.895 (95% CI: 0.854–0.937), an AUPRC of 0.796 (95% CI: 0.730–0.851), a sensitivity of 0.874 (95% CI: 0.810–0.938), a specificity of 0.754 (95% CI: 0.709–0.799), a PPV of 0.511 (95% CI: 0.438–0.585), and an NPV of 0.953 (95% CI: 0.928–0.978).

## 4. Discussion

In this retrospective study, the AI-ECG model identified LVSD with high diagnostic accuracy in patients presenting to the ED and retained good discrimination in those with hypotension or an elevated shock index. Importantly, these findings extend the existing evidence by demonstrating preserved model performance in acutely ill ED patients, a population in whom early recognition of LVSD may have immediate diagnostic, therapeutic, and prognostic relevance.

Early identification of LVSD in the ED may have clinical value beyond diagnostic classification alone. LVSD reflects impaired cardiac reserve and may signal a higher-risk clinical trajectory, particularly in patients presenting with hypotension, shock, respiratory distress, or other manifestations of acute decompensation. During the initial phase of ED care, clinicians must often make time-sensitive decisions regarding intravenous fluid administration, vasopressor or inotropic support, cardiac monitoring, intensive care unit disposition, and the need for urgent cardiology consultation or echocardiographic assessment [18,19]. Failure to recognize LVSD may contribute to inappropriate fluid loading, delayed identification of cardiogenic shock, or underestimation of short-term clinical risk [20]. Conversely, rapid detection of LVSD may help identify patients who require closer monitoring, earlier escalation of care, and more targeted hemodynamic management. Although the present study was not designed to determine whether AI-ECG-guided care improves clinical outcomes, the rapid detection of LVSD in hemodynamically unstable ED patients suggests that this approach may support early risk stratification and therapeutic prioritization in a population at increased risk of adverse outcomes, including clinical deterioration and mortality [21,22].

Point-of-care echocardiography is an established and increasingly standardized bedside method for evaluating cardiac function in the ED [10]. It provides direct structural and functional information and remains essential for confirming cardiac dysfunction. However, its use depends on operator expertise, the availability of equipment and trained personnel, and adequate image-acquisition conditions, all of which may be limited in crowded or time-sensitive emergency settings. AI-ECG analysis may serve a distinct but complementary role. Because it uses a standard 12-lead ECG that is routinely obtained in most ED patients, requires no additional imaging procedure, and generates an automated result within seconds, it may help identify patients who warrant focused echocardiographic confirmation. Accordingly, AI-ECG analysis should not be regarded as a substitute for point-of-care echocardiography, but rather as a rapid first-pass screening tool that may facilitate earlier risk recognition and prioritization of echocardiographic assessment. Consistent with this screening role, the predefined threshold favors sensitivity. The high negative predictive value (0.982) indicates that a low score makes LVSD unlikely and may therefore help deprioritize urgent echocardiography when LVSD is the primary clinical concern, whereas the moderate positive predictive value (0.415) indicates that a positive result should prompt echocardiographic confirmation rather than being interpreted as establishing a diagnosis of LVSD.

The ED environment also poses important challenges for ECG-based analysis. ECGs obtained in this setting may be affected by multiple clinical and technical factors, including patient movement, pain, discomfort, muscle rigidity, anxiety, and other sources of noise or artifact [23]. Previous studies have indicated that standardized ECG acquisition is important for reliable algorithm performance and that variation in precordial lead position or placement technique may introduce additional variability into ECG recordings [24]. In the present study, ECGs were acquired by a heterogeneous group of healthcare personnel, including interns, residents, nurses, and paramedics, which may have affected recording quality and consistency. Acute conditions such as respiratory distress may also influence both cardiac function and ECG findings [25]. To further characterize the clinical context, we assessed chest radiographic abnormalities, which were present in 54.9% of patients with LVSD and 21.8% of those without LVSD. Despite these multiple potential sources of variability, the high diagnostic accuracy observed in this study is noteworthy and suggests that AI-ECG analysis may retain clinical utility under the challenging conditions of emergency care.

The performance of the AI-ECG model in patients with abnormal vital signs requires cautious interpretation. Among patients with a shock index ≥ 0.9, the model retained good discrimination (AUROC, 0.895; 95% CI: 0.854–0.937), operating at a level only modestly lower than that observed in the overall cohort (AUROC, 0.934; 95% CI: 0.923–0.945). This finding is noteworthy because acute hemodynamic compromise can alter both cardiac function and ECG characteristics [25], potentially reducing algorithm performance. The preserved discrimination therefore suggests that model performance remained stable under these conditions, although the electrocardiographic features underlying this performance were not directly examined. Findings in the hypotensive subgroup were directionally consistent (AUROC, 0.885; 95% CI: 0.786–0.984); however, this subgroup included only 75 patients and 10 LVSD events. Given the resulting wide confidence interval, these estimates should be considered exploratory and hypothesis-generating rather than confirmatory. Validation in larger cohorts of hemodynamically unstable patients is therefore warranted.

In the present study, the ECG and reference echocardiogram were not obtained concurrently because echocardiography could be performed at any time within 30 days of the ECG. This interval is consistent with that used in the development study of this class of algorithms, in which ECG and echocardiography were paired within a comparable 30-day window [3]. Nevertheless, most examinations in our cohort were performed close to ED presentation, with 67.8% completed within 3 days and 86.6% within 7 days. The interval was shortest among patients with LVSD, 81.4% of whom underwent echocardiography within 3 days. Longer intervals were therefore concentrated among patients without LVSD, in whom interval changes in ejection fraction would be less likely to alter the reference classification. Although residual reference-standard misclassification cannot be excluded, its effect on the estimated diagnostic performance is likely to have been limited.

This study represents external validation conducted at a single tertiary care center. Because echocardiography was performed selectively and the cohort was restricted to patients who underwent both ECG and echocardiography, the observed LVSD prevalence of 11.7% reflects a population enriched for cardiac disease. This prevalence lies at the upper end of the range reported in the development and validation cohorts for this class of algorithms, in which reduced ejection fraction occurred in approximately 4–14% of patients across teaching and community hospitals [3,12]. This case mix has important implications for interpretation of the performance estimates. Unlike predictive values, the AUROC is not directly determined by disease prevalence; however, discrimination may still vary according to patient case mix and clinical setting, as prior development studies have shown that performance differs according to the characteristics of the validating institution [3]. Predictive values are prevalence-dependent. Assuming comparable sensitivity and specificity, the negative predictive value would be expected to increase in lower-prevalence populations, whereas the positive predictive value would decline and should therefore be interpreted as specific to a cohort enriched for cardiac disease, such as the present cohort. Validation in community and secondary-care settings is needed before the findings can be generalized more broadly.

This study has several limitations. First, the single-center design may have introduced selection bias related to the characteristics of our institution. In addition, echocardiography was performed at the discretion of the treating physician rather than systematically, and the clinical indication was not documented in a retrievable form. Second, the model was developed and validated within a single national population, and its performance in other ethnic populations remains to be established. These limitations, together with the retrospective design, underscore the need for prospective, multicenter validation across diverse populations and clinical settings.

## 5. Conclusions

In this study, the AI-ECG model identified LVSD with high diagnostic accuracy in patients presenting to the ED and retained good discrimination among patients with an elevated shock index, with directionally consistent but exploratory findings in the smaller hypotensive subgroup. Because AI-ECG analysis requires only a standard 12-lead ECG and is less dependent on operator expertise than point-of-care echocardiography, it may serve as a complementary screening tool to help prioritize echocardiographic assessment. Whether implementation of this approach improves patient outcomes requires prospective evaluation.

## Figures and Tables

**Table 1:** Baseline characteristics.

	Non-LVSD (*n* = 3998)	LVSD (*n* = 531)	*p*-Value
**Demographics**			
Age, years	68.0 [58.0–78.0]	73.0 [60.0–81.0]	<0.001
Male sex	2411 (60.3)	343 (64.6)	0.057
**Comorbidities**			
Hypertension	1727 (43.2)	260 (49.0)	0.012
Diabetes mellitus	991 (24.8)	192 (36.2)	<0.001
Cardiovascular disease	737 (18.4)	185 (34.8)	<0.001
Chronic kidney disease	170 (4.3)	46 (8.7)	<0.001
Stroke	206 (5.2)	27 (5.1)	0.947
**Presentation**			
Chief complaint			<0.001
Cardiovascular	1568 (39.2)	202 (38.0)	
Respiratory	454 (11.4)	217 (40.9)	
Neurologic	1136 (28.4)	25 (4.7)	
General	513 (12.8)	60 (11.3)	
Other	327 (8.2)	27 (5.1)	
Cardiac arrest	14 (0.4)	4 (0.8)	0.165
Hypotension	65 (1.6)	10 (1.9)	0.662
**Vital signs**			
SBP, mmHg	151.0 [130.0–171.0]	140.0 [122.0–160.0]	<0.001
DBP, mmHg	86.0 [74.0–98.0]	85.0 [71.0–99.0]	0.607
Heart rate, bpm	84.0 [72.0–98.0]	97.0 [80.0–112.0]	<0.001
Respiratory rate, /min	18.0 [18.0–18.0]	20.0 [18.0–26.0]	<0.001
SpO_2_, %	98.0 [97.0–98.0]	98.0 [96.0–99.0]	0.050
Body temperature, °C	36.7 [36.3–37.0]	36.5 [36.1–36.9]	<0.001
**Laboratory and radiographic findings**			
NT-proBNP, pg/mL	359.1 [74.8–1732.2]	4601.1 [1806.4–10,972.5]	<0.001
Abnormal chest radiograph	754 (21.8)	270 (54.9)	<0.001
**Diagnosis and echocardiography**			
Heart disease as final ED diagnosis	1709 (42.7)	383 (72.1)	<0.001
LVEF, %	58.0 [55.0–61.0]	32.0 [26.0–36.0]	<0.001
Admission-to-echo interval, days	2.0 [1.0–5.0]	1.0 [0.0–3.0]	<0.001
Interval category			<0.001
Same day	737 (18.4)	146 (27.5)	
1–3 days	1902 (47.6)	286 (53.9)	
4–7 days	798 (20.0)	55 (10.4)	
>7 days	561 (14.0)	44 (8.3)	

Categorical variables are presented as n (%), and continuous variables as medians [interquartile ranges]. LVSD, left ventricular systolic dysfunction; ED, emergency department; SBP, systolic blood pressure; DBP, diastolic blood pressure; SpO_2_, peripheral oxygen saturation; NT-proBNP, N-terminal pro-B-type natriuretic peptide; LVEF, left ventricular ejection fraction.

**Table 2:** Diagnostic performance of the AI-ECG model.

	AUROC	AUPRC	Sensitivity	Specificity	PPV	NPV
AI-ECG model	0.934 (0.923–0.945)	0.735 (0.661–0.754)	0.883 (0.856–0.911)	0.835 (0.823–0.846)	0.415 (0.386–0.444)	0.982 (0.977–0.986)

All estimates are presented with 95% confidence intervals in parentheses. AI-ECG, artificial intelligence-based electrocardiogram; AUROC, area under the receiver operating characteristic curve; AUPRC, area under the precision–recall curve; PPV, positive predictive value; NPV, negative predictive value.

**Table 3:** Diagnostic performance of the AI-ECG model in patient subgroups stratified by vital signs.

	AUROC	AUPRC	Sensitivity	Specificity	PPV	NPV
**SBP**						
SBP < 90 (*n* = 75)	0.885 (0.786–0.984)	0.633 (0.383–0.843)	0.900 (0.714–1.00)	0.708 (0.597–0.818)	0.321 (0.148–0.494)	0.979 (0.937–1.00)
SBP ≥ 90 (*n* = 4420)	0.935 (0.924–0.947)	0.739 (0.707–0.768)	0.881 (0.853–0.909)	0.838 (0.827–0.850)	0.417 (0.388–0.447)	0.982 (0.977–0.986)
**Shock index**						
Shock index < 0.9 (*n* = 4042)	0.939 (0.929–0.950)	0.720 (0.686–0.752)	0.883 (0.853–0.914)	0.844 (0.832–0.856)	0.397 (0.365–0.428)	0.984 (0.980–0.989)
Shock index ≥ 0.9 (*n* = 453)	0.895 (0.854–0.937)	0.796 (0.730–0.851)	0.874 (0.810–0.938)	0.754 (0.709–0.799)	0.511 (0.438–0.585)	0.953 (0.928–0.978)

All estimates are presented with 95% confidence intervals in parentheses. AI-ECG, artificial intelligence-based electrocardiogram; AUROC, area under the receiver operating characteristic curve; AUPRC, area under the precision–recall curve; PPV, positive predictive value; NPV, negative predictive value; SBP, systolic blood pressure.

## Data Availability

The data presented in this study are available upon reasonable request from the corresponding author. The data are not publicly available due to privacy and ethical restrictions.

## References

[1] Metra M., Teerlink J.R. (2017). Heart failure. Lancet.

[2] Galasko G.I., Barnes S.C., Collinson P., Lahiri A., Senior R. (2006). What is the most cost-effective strategy to screen for left ventricular systolic dysfunction: Natriuretic peptides, the electrocardiogram, hand-held echocardiography, traditional echocardiography, or their combination?. Eur. Heart J..

[3] Cho J., Lee B., Kwon J.-M., Lee Y., Park H., Oh B.-H., Jeon K.-H., Park J., Kim K.-H. (2021). Artificial intelligence algorithm for screening heart failure with reduced ejection fraction using electrocardiography. ASAIO J..

[4] Adedinsewo D., Carter R.E., Attia Z., Johnson P., Kashou A.H., Dugan J.L., Albus M., Sheele J.M., Bellolio F., Friedman P.A. (2020). Artificial intelligence-enabled ECG algorithm to identify patients with left ventricular systolic dysfunction presenting to the emergency department with dyspnea. Circ. Arrhythmia Electrophysiol..

[5] Kwon J.-M., Jo Y.-Y., Lee S.Y., Kang S., Lim S.-Y., Lee M.S., Kim K.-H. (2022). Artificial intelligence-enhanced smartwatch ECG for heart failure-reduced ejection fraction detection by generating 12-lead ECG. Diagnostics.

[6] Attia Z.I., Kapa S., Lopez-Jimenez F., McKie P.M., Ladewig D.J., Satam G., Pellikka P.A., Enriquez-Sarano M., Noseworthy P.A., Munger T.M. (2019). Screening for cardiac contractile dysfunction using an artificial intelligence-enabled electrocardiogram. Nat. Med..

[7] Grün D., Rudolph F., Gumpfer N., Hannig J., Elsner L.K., von Jeinsen B., Hamm C.W., Rieth A., Guckert M., Keller T. (2020). Identifying heart failure in ECG data with artificial intelligence—A meta-analysis. Front. Digit. Health.

[8] McDonagh T.A., Metra M., Adamo M., Gardner R.S., Baumbach A., Böhm M., Burri H., Butler J., Čelutkienė J., Chioncel O. (2021). 2021 ESC Guidelines for the diagnosis and treatment of acute and chronic heart failure. Eur. Heart J..

[9] Wang T.J., Evans J.C., Benjamin E.J., Levy D., LeRoy E.C., Vasan R.S. (2003). Natural history of asymptomatic left ventricular systolic dysfunction in the community. Circulation.

[10] Alerhand S., Adrian R.J., Taylor L.A. (2024). Cardiac point-of-care ultrasound: An emergency medicine review. Emerg. Med. Clin. N. Am..

[11] Bjerkén L.V., Rønborg S.N., Jensen M.T., Ørting S.N., Nielsen O.W. (2023). Artificial intelligence enabled ECG screening for left ventricular systolic dysfunction: A systematic review. Heart Fail. Rev..

[12] Lee M.S., Jang J.-H., Kang S., Han G.I., Yoo A.-H., Jo Y.-Y., Son J.M., Kwon J.-M., Lee S., Lee J.S. (2025). Transparent and robust artificial intelligence-driven electrocardiogram model for left ventricular systolic dysfunction. Diagnostics.

[13] Pandey A., Keshvani N., Segar M.W., Kwon J.-M., Lee H.S., Bhograj C., Mashilane K.I., Jain N., Mwiti W., Wambari E. (2026). Artificial intelligence electrocardiogram and left ventricular systolic dysfunction in Kenya. JAMA Cardiol..

[14] Grafstein E., Bullard M.J., Warren D., Unger B. (2008). Revision of the Canadian Emergency Department Information System (CEDIS) Presenting Complaint List version 1.1. Can. J. Emerg. Med..

[15] Kwon J.-M., Kim K.-H., Jeon K.-H., Kim H.M., Kim M.J., Lim S.-M., Song P.S., Park J., Choi R.K., Oh B.-H. (2019). Development and validation of deep-learning algorithm for electrocardiography-based heart failure identification. Korean Circ. J..

[16] Lee Y., Choi B., Lee M.S., Jin U., Yoon S., Jo Y.-Y., Kwon J.-M. (2022). An artificial intelligence electrocardiogram analysis for detecting cardiomyopathy in the peripartum period. Int. J. Cardiol..

[17] Koch E., Lovett S., Nghiem T., Riggs R.A., Rech M.A. (2019). Shock index in the emergency department: Utility and limitations. Open Access Emerg. Med..

[18] Myburgh J.A., Mythen M.G. (2013). Resuscitation fluids. N. Engl. J. Med..

[19] Graham C.A., Parke T.R. (2005). Critical care in the emergency department: Shock and circulatory support. Emerg. Med. J..

[20] Long B., Koyfman A., Gottlieb M. (2018). Management of heart failure in the emergency department setting: An evidence-based review of the literature. J. Emerg. Med..

[21] Jones N.R., Roalfe A.K., Adoki I., Hobbs F.D.R., Taylor C.J. (2019). Survival of patients with chronic heart failure in the community: A systematic review and meta-analysis. Eur. J. Heart Fail..

[22] Cavefors O., Holmqvist J., Bech-Hanssen O., Einarsson F., Norberg E., Lundin S., Omerovic E., Ricksten S.-E., Redfors B., Oras J. (2021). Regional left ventricular systolic dysfunction associated with critical illness: Incidence and effect on outcome. ESC Heart Fail..

[23] Littmann L. (2021). Electrocardiographic artifact. J. Electrocardiol..

[24] Harrigan R.A. (2006). Electrode misconnection, misplacement, and artifact. Emerg. Med. Clin. N. Am..

[25] Cross T.J., Kim C.H., Johnson B.D., Lalande S. (2020). The interactions between respiratory and cardiovascular systems in systolic heart failure. J. Appl. Physiol..

